# Metabolic dysfunction-associated steatotic liver disease, biomarkers, and cardiovascular risk factors in children with excess weight

**DOI:** 10.1016/j.jped.2026.101534

**Published:** 2026-04-01

**Authors:** Elisabeth A. Machado, Beatriz L.C. Themistocles, Jorge M.R. Teixeira, Fernanda M.G. Jannuzzi, Geísa Tomaz, Cecilia L. Oliveira, Isabel R. Madeira, Carlos Terra, Maria das Graças C. Souza, Alexandra Monteiro, Eliete Bouskela, Marise E. Marsillac, Paulo F. Collett-Solberg

**Affiliations:** a Universidade do Estado do Rio de Janeiro, Instituto de Aplicação Fernando Rodrigues da Silveira, Rio de Janeiro, RJ, Brazil; b Universidade do Estado do Rio de Janeiro, Centro Biomédico, Laboratório de Pesquisa Clínica e Experimental em Biologia Vascular, Rio de Janeiro, RJ, Brazil; c Universidade do Estado do Rio de Janeiro, Postgraduate Program in Clinical and Experimental Physiopathology, Rio de Janeiro, RJ, Brazil; d Universidade do Estado do Rio de Janeiro, Instituto de Nutrição, Rio de Janeiro, RJ, Brazil; e Universidade do Estado do Rio de Janeiro, Faculdade de Ciências Médicas, Department of Internal Medicine, Endocrinology Care Teaching Unit, Rio de Janeiro, RJ, Brazil; f Universidade do Estado do Rio de Janeiro, Faculdade de Ciências Médicas, Department of Pediatrics, Rio de Janeiro, RJ, Brazil; g Universidade do Estado do Rio de Janeiro, Gastroenterology Department, Hepatology Unit, Rio de Janeiro, RJ, Brazil; h Universidade do Estado do Rio de Janeiro, Faculdade de Ciências Médicas, Department of Radiology, Rio de Janeiro, RJ, Brazil; i Universidade do Estado do Rio de Janeiro, Faculdade de Ciências Médicas, Department of Internal Medicine, Discipline of Endocrinology, Rio de Janeiro, RJ, Brazil

**Keywords:** Transient elastography, Pediatric obesity, Adiponectin, Insulin, MASLD

## Abstract

**Objectives:**

The increasing prevalence of metabolic dysfunction associated with steatotic liver disease (MASLD) is consistent with the global obesity epidemic. The aim of the study was to investigate the presence of MASLD in a high-risk group of children and to compare cardiovascular risk factors between the groups with and without MASLD, in addition to investigating potential biomarkers.

**Methods:**

This cross-sectional study was carried out at a public institution with thirty-eight patients (19 girls, 13.2 ± 2.7 y) with excess weight. Blood pressure (BP), abdominal fat, carotid intima-media thickness (CIMT), and blood biochemistry were evaluated. Steatosis was assessed by the controlled attenuation parameter (CAP) obtained via transient elastography, and the participants were grouped as ¨Non-Steatosis¨ (*n* = 24) and ¨Steatosis¨ (*n* = 14).

**Results:**

The “Steatosis group” showed higher BP (Z SBP 1.56 *vs.* -0.25; Z DBP 1.38 *vs*. 0.07; *p* = 0.0001), BMI (z-BMI 3.48 *vs*. 2.39; *p* < 0.0001), right CIMT (0.07 *vs.* 0.06 cm; *p* = 0.013), basal insulin (41 *vs*. 14 mcUI/l; *p* < 0.0001), and HOMA-IR (8.2 *vs*. 3.1; *p* < 0.0001), and lower adiponectin (2.75 *vs.* 6.46 µg/ml; *p* = 0.004) compared to the “Non-Steatosis” group even after z-BMI adjustments. ROC curves indicated adiponectin (cutoff ≤ 4.5 μg/mL) and insulin (cutoff ≥ 22 mcIU/mL) as biomarkers of steatosis.

**Conclusion:**

In pediatric age with excess weight, hepatic steatosis was associated with higher BP, severe obesity, and worse glycemic profile. Low adiponectin and high insulin levels appeared as possible biomarkers of MASLD. The study reinforces the need for children with obesity and a worse metabolic profile to be investigated for early diagnosis of MASLD.

## Introduction

As a multifactorial disease, obesity is considered a worldwide syndemic problem in all age groups, and a risk factor for several conditions, such as metabolic dysfunction associated with steatotic liver disease (MASLD) [1]. The high prevalence of both diseases has been driven by increased sedentary behavior, excessive calorie intake from unbalanced and unhealthy diets, socioeconomic status, and genetic and epigenetic factors [2]. It is noted that a recent meta-analysis reported that the prevalence of MASLD in children is between 5 % and 10 % in the general population, and can be detected in 47 % of children with obesity, mainly affecting boys [3].

Current understanding of MASLD (fatty infiltration in more than 5 % of hepatocytes) in the pediatric age group is that untreated metabolic dysfunction can lead to worsening liver injury. Similar to the disease trajectory observed in adults, without appropriate intervention, there is a risk of progression to steatohepatitis (MASH) with inflammation and fibrosis, and in late adolescence or early adulthood, developing cirrhosis [4]. Furthermore, MASLD is also related to the increased risk of cardiovascular diseases, nowadays the major cause of death in this group [5].

The pathogenesis of MASLD is multifactorial, including genetic and environmental factors such as diet with elevated content of refined sugar and saturated fat, which act together to increase hepatic fat deposition. The excess of fat stimulates mitochondrial dysfunction, induces oxidative stress, and apoptosis [6]. Beyond insulin resistance, the lipotoxicity due to obesity can also contribute to the development of liver inflammation. Furthermore, the local and systemic production of pro-inflammatory cytokines may play a key role in the progression to steatohepatitis [7].

The gold standard to diagnosing MASLD is the hepatic biopsy, which is difficult and stressful to perform in children. The hepatic transient elastography is a less invasive method that can analyze the presence of fibrosis (liver stiffness measurement or LSM) and steatosis (controlled attenuation parameter or CAP) [8]. It is well studied in the adult population, although pediatric data are scarce. Given this gap in the literature, the present study evaluated the possible differences in obesity markers and cardiovascular risk factors among children and adolescents with excess weight in the presence or absence of steatosis according to transient hepatic elastography, in addition to evaluating their correlation with CAP and LSM, and investigating possible biomarkers for MASLD.

## Methods

### Subjects

This cross-sectional study evaluated 38 patients (19 girls) aged 5 to 17 years, diagnosed with overweight or obesity. Outpatient monitoring of participants is carried out by the APOIO (Ambulatório de Pesquisa em Obesidade Infantil) project, which offers a lifestyle change-based intervention by a multidisciplinary team from a public university hospital in Rio de Janeiro (RJ, Brazil). Nutritional status was defined according to the WHO standardized BMI Z-Score (z-BMI) for age and sex (> + 2 z-scores for obesity; > + 1 and ≤ + 2 for overweight; ≥ - 2 and ≤ +1 for normal weight), calculated using WHO Antro Plus™software version 1.04 (WHO, Geneva, Switzerland).

To increase the number of volunteers with steatosis, the inclusion criteria for the study population comprised characteristics that placed them at high risk for MASLD and consisted of: a) a previous abdominal ultrasonography showing hepatic steatosis; or b) alanine aminotransferase (ALT) ≥ 22 U/L for girls and ≥ 26 U/L for boys; or c) a relation aspartate aminotransferase (AST)/ALT > 1 [9]. The exclusion criteria were the presence of genetic, kidney, hematological, hepatic, rheumatologic, cardiovascular, respiratory, and endocrine diseases; hepatitis; any condition that would affect weight gain or metabolic function; and consumption of alcoholic beverages. All participants and their legal guardians provided written informed consents. The study received approval from the local Ethics and Research Committee (CAAE: 00,400,318.9.0000.5259).

**Figure 1:**
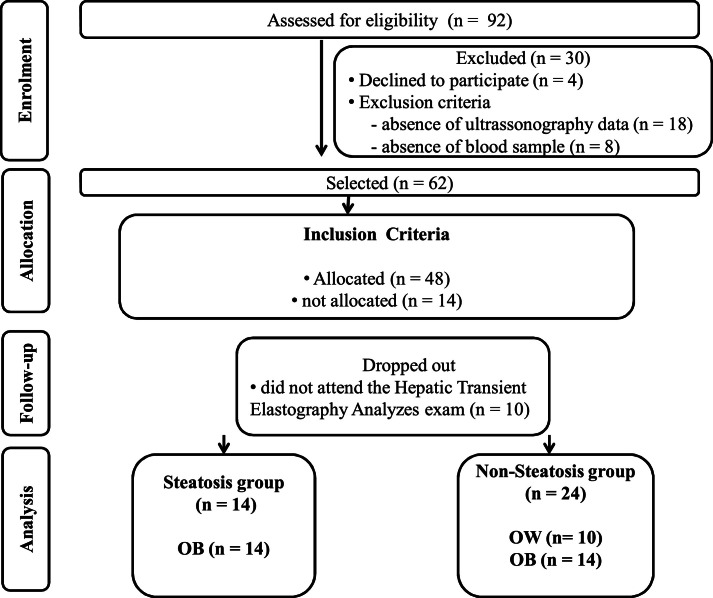
Flow diagram of study.

Figure 1 summarizes the procedures for the sample selection, depicting the number of participants after applying the inclusion criteria and the stratification according to hepatic transient elastography.

### Experimental design

Data collection occurred between November 2018 and November 2019. On the first visit, patients underwent clinical and nutritional assessments. On the second visit (the next day or up to a week later), a fasting blood sample was collected, followed by abdominal and carotid ultrasound assessments at visit 3, and hepatic transient elastography analyses at visit 4.

The participants were grouped as follows: 40 % (*n* = 14) were allocated in the "Steatosis group" and 60 % (*n* = 24) to the "Non-Steatosis" group".

### Clinical assessments

Blood pressure (BP) was assessed with a Tycos sphygmomanometer (Welch Allyn Company, Arden, NC. USA) in a sitting position, following the standard protocol [10]. The anthropometric data were collected barefoot and wearing light clothes. Weight was assessed using a digital scale (Filizola®, São Paulo, SP, Brazil) to the nearest 0.1 Kg. Height was measured with a wall-mounted stadiometer (Tonelli®, Criciúma, SC, Brazil) to the nearest 0.1 cm. Abdominal circumference (AC) was measured immediately above the iliac crest, while waist circumference (WC) was measured at the midpoint between the last costal arch and the iliac crest [11]. All circumferences were measured with a non-distensible tape (nearest 0.1 cm). The daily physical activity was estimated by the Physical Activity Questionnaire for Older Children (PAQ-C) which quantifies physical activity performed in the last seven days through questions rated by a 5-point scale (1 = did not practice; 5 = practiced every day of the week) and the final score corresponded to the average of all responses (1- very sedentary; 2- sedentary; 3- moderately active; 4- active; 5- very active [12].

### Laboratorial analyzes

Blood samples were collected in the morning after 12 h fasting. Samples for adiponectin, TNF- α and leptin evaluations were centrifuged and immediately conserved at -80 °C for later analysis (commercial ELISA kits for adiponectin, leptin, from R & D Systems, Minneapolis, MN, EUA). Insulin levels were measured using the kit Elecsys Insulin (Roche Diagnostics, Indianapolis, IN, USA), and insulin resistance was assessed by the HOMA-IR and HOMA-ß equations. Regular biochemical analyses were performed for triglycerides (TG), ALT, AST, gamma glutamyl transferase (GGT), and ferritin.

### Ultrasonography analyzes

All ultrasounds were performed by a single experienced investigator. The equipment was an Aplio XG ultrasound machine, model SSA-790A (Toshiba Medical Systems, Tokyo, Japan). The abdominal fat was divided into subcutaneous adipose tissue (SAT) – the distance between the skin and the anterior face of the alba line, and the visceral adipose tissue (VAT), corresponding to the distance between the posterior face of the alba line and the anterior wall of the abdominal aorta artery [13].

The carotid intima media thickness (CIMT) was defined by the distance between the endothelium and the luminal and distal muscle layer, evaluated at the common carotid artery 1.5 to 2 cm below the bifurcation, and values above 0.045 cm were classified as elevated [14].

### Hepatic transient elastography analyzes

The transient elastography was performed by an experienced examiner with a Fibroscan device (Echosens, Paris, France), that consist in an 5 MHz ultrasound transducer placed on the shaft of a vibrator, through which the values of CAP (controlled attenuation parameter) and LSM (liver stiffness measurement) are assessed. Both M or XL probes were used, with the XL being preferred for children with obesity. The exam was performed after 2 hours of fast assessing the right lobe of the liver in the intercostal space while individuals are lying in a supine position with the right arm in maximum abduction.

The LSM is estimated from the velocity at which the shear waves cross through hepatic tissues; the velocity of the wave is directly related to the stiffness, expressed in kilopascal (kPa), which corresponds to the degree of fibrosis or its absence. The cut-off values used are F0-F1 (until 7 kPa), F2 (7 – 8.7 kPa), F3 (8.7 -10.3 kPa), and F4 (> 10.3 kPa), considering that sensitivity is 97 % and specificity of 91 % [15].

The presence of hepatic steatosis is assessed by the measure of CAP and expressed in decibel/meters – dB/m. It is evaluated using the same principles as stiffness, the attenuation of hepatic tissue to the radiofrequency, go and come waves, which estimates the value of CAP and then the steatosis degree. The presence or not of steatosis can be classified as: S0 (100 - 247 dB/m and < 5 – 10 % hepatocytes), S1 (248– 267 dB/m and 6 -11 % – 33 %), S2 (268 – 280 dB/m and 34 – 66 %) and S3 (> 280 dB/m and > 66 % of hepatocytes affected). In this study, the cut-off of 248 dB/m (S1) was used to define the presence or absence of steatosis.

The steatosis sensibility varies according to the percentage of affected hepatocytes, ranging from 76 – 85 % with a specificity of 79 %, in adults, and the median value of 10 successful acquisitions, defined as acquisitions with at least a 60 % success rate and with an interquartile range of less than 20, is used as representative of liver stiffness and final CAP value.

### Statistical analyzes

Data normality was assessed by the Shapiro-Wilk test. Results were depicted as mean and standard deviation (SD) for normal distribution, and as median and interquartile interval (IQ25–75 %) for non-normal distribution.

The comparison between the subgroups with and without steatosis was analyzed by Student's *t*-test for independent samples or Mann-Whitney (nonparametric) for numerical data, and by the chi-square test or Fisher's exact test for categorical data. Receiver Operating Characteristic (ROC) curves were used to try to assess the diagnosis of steatosis. The association between anthropometric, metabolic, hepatic, inflammatory, hormonal, ultrasound, and transient hepatic elastography measurements was analyzed using Spearman's correlation coefficient. Binary logistic regression was used to verify the relationship of the variables with steatosis adjusted for z-BMI.

The criterion for determining significance adopted was the 5 % level (*p* < 0.05). The statistical analysis was processed using SPSS software version 26.

## Results

The characteristics of the sample are presented in Table 1. The proportion of girls and boys was similar (19 female and 19 male). Participants mostly presented obesity (*N* = 28; 73.7 %), were already in puberty (*N* = 30; 78.9 %) and reported being moderately active (*N* = 22; 57.9 %).

**Table 1:** Anthropometrical data, blood pressure, transient elastography, ultrasound and biochemical values and characteristics of total sample.

Variable	n	mean	SD	median	IQ			minimum	maximum
Age	38	13.2	2.7					7	17.9
BMI Z-Score	38	2.79	0.87					1.15	4.64
AC (cm)	26	98.1	15.9					77	136
WC (cm)	28	95.9	18.6					71	145
Z SBP *	38			0.30	-0.89	-	1.30	-2.03	5.79
Z DBP *	38			0.39	-0.03	-	1.33	-0.98	3.23
LSM (kPa)	38	5.38	1.25					3.4	10.2
CAP (dB/m)	38	214	66					103	400
CIMT right (cm)	36	0.06	0.01					0.04	0.10
CIMT left (cm)	36	0.06	0.01					0.04	0.08
SAT (cm)	35	3.60	1.41					1.4	6.5
VAT (cm)	36	4.62	1.75					2.1	10.1
Insulin (mcUI/mL) *	38			18	12	-	36	9.1	133
HOMA-IR *	38			4.1	2.6	-	7.9	1.7	34.1
HOMA-ß *	38			317	199	-	656	106	1500
TG (mg/dl) *	38			97	75	-	134	42	407
AST (UI/L) *	37			22	20	-	27	13	64
ALT (UI/L) *	37			21	15.5	-	34	5	96
GGT (UI/L) *	37			23	17.5	-	30.5	10	53
Ferritin (ng/mL) *	34			46	28	-	76	15	303
Adiponectin (µg/mL) *	38			4.72	2.68	-	7.62	1.77	15.09
Leptin (ng/mL) *	38			36	20	-	62	11	96

BMI Z-Score, body mass index Z-score; AC, abdominal circumference; WC, waist circumference; SBP, systolic blood pressure; DBP, diastolic blood pressure; LSM, liver stiffness measurement; CAP, controlled attenuation parameter; CIMT, carotid intima-media thickness; SAT, subcutaneous abdominal tissue; VAT, visceral abdominal tissue; HOMA, homeostatic model assessment; IR, insulin resistance; TG, triglycerides; AST, aspartate aminotransferase; ALT, alanine aminotransferase; GGT, gamma glutamyl transferase.

⁎ Non-Gaussian distribution.

Table 2 shows "Steatosis group" *vs. “*Non-Steatosis group" based on transient elastography analyses. Steatosis was classified according to the degree of involvement and CAP value: S3 (15.8 %. *N* = 6), S2 (18.4 %. *N* = 7) and S1 (2.7 %. *N* = 1). Pubertal status, sex and physical activity level were not different between groups. Obesity was present in all patients of the "Steatosis group" and in 60 % of "Non-Steatosis group". The presence of fibrosis was investigated by LSM and was detected in only three (7.9 %) participants of the "Steatosis group" (two staged as F2 and one as F3).

**Table 2:** Comparison between the “steatosis” and “non-steatosis” groups before and after z-BMI adjustment regarding anthropometric, blood pressure, elastography, ultrasound and biochemical data.

Variable	Steatosis (*n* = 14)				Non-Steatosis (*n* = 24)				*p value*^a^	*p value*^b^
Age		13.8	±	2.4		12.9	±	2.8	0.32	0.28
Z BMI		3.48	±	0.52		2.39	±	0.79	**< 0.0001**	
AC (cm)		111.9	±	15.6		90.7	±	10.5	**0.0003**	0053
WC (cm)		112.2	±	18.7		86.9	±	10.9	**0001**	**0050**
Z SBP *	1.56	0.44	-	3.86	-0.25	-1.17	-	0.67	**0.0001**	**0030**
Z DBP *	1.38	1.16	-	2.60	0.07	-0.38	-	0.46	**< 0.0001**	**0017**
LSM (kPa)		6.16	±	1.02		4.93	±	0.80	**0004**	**0035**
CAP (dB/m)		288	±	43		170	±	29	**< 0.0001**	0.99
CIMT right (cm)		0.07	±	0.01		0.06	±	0.01	**0013**	**0046**
CIMT left (cm)		0.06	±	0.01		0.06	±	0.01	0.12	0052
SAT (cm)		4.53	±	1.37		3.05	±	1.13	**0001**	0.29
VAT (cm)		5.94	±	1.67		3.87	±	1.32	**0.0002**	0051
Insulin (mcUI/mL) *	41	22	-	57	14	10	-	20	**< 0.0001**	**0036**
HOMA-IR*	8.2	4,5	-	14.3	3.1	2.2	-	4.5	**< 0.0001**	**0050**
HOMA-ß*	664	304	-	907	268	161	-	351	**0003**	0.13
TG (mg/dl) *	123	93	-	167	85	60	-	107	**0012**	0.33
AST (UI/L) *	23.5	22	-	31.5	21	18	-	25	0061	0.17
ALT (UI/L) *	33	24	-	43	19	13	-	24	**0002**	**0037**
GGT (UI/L) *	26	22	-	36	21	16	-	29	**0024**	0.13
Ferritin (ng/mL) *	74	45	-	142	42	27	-	57	**0012**	0089
Adiponectin (µg/mL) *	2.75	2.07	-	3.56	6.46	4.67	-	8.60	**0.0004**	**0010**
Leptin (ng/mL) *	57	35	-	91	29	14	-	40	**0001**	0.31

BMI Z-Score, body mass index Z-score; AC, abdominal circumference; WC, waist circumference; SBP, systolic blood pressure; DBP, diastolic blood pressure; LSM, liver stiffness measurement; CAP, controlled attenuation parameter; CIMT, carotid intima-media thickness; SAT, subcutaneous abdominal tissue; VAT, visceral abdominal tissue; HOMA, homeostatic model assessment; IR, insulin resistance; TG, triglycerides; AST, aspartate aminotransferase; ALT, alanine aminotransferase; GGT, gamma glutamyl transferase.

⁎ Non – Gaussians distribution.

a Student's *t*-test for independent samples or Mann-Whitney.

b Individual logistic regression analysis for adjusted by BMI Z-score.

**Figure 2:**
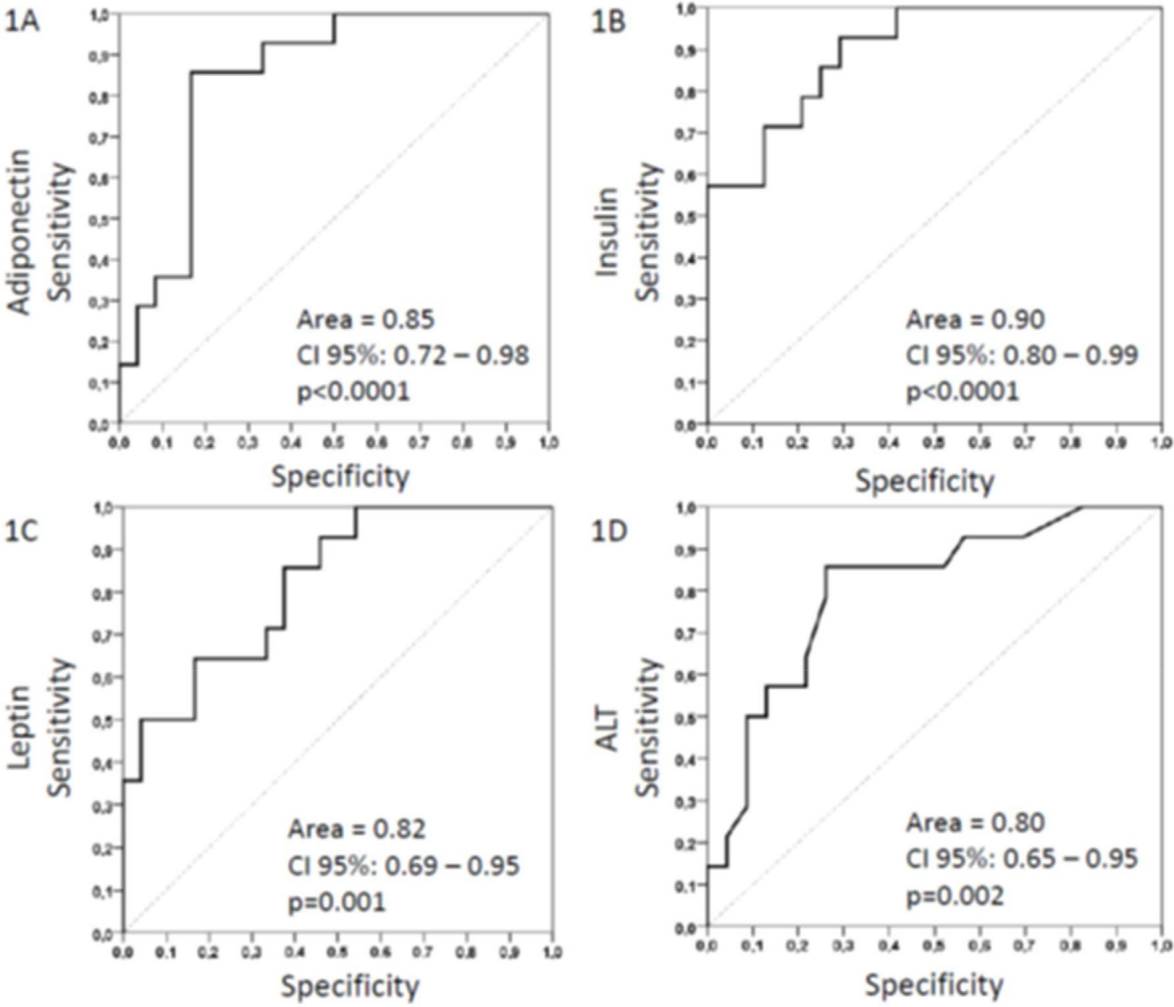
ROC curves for predict steatosis. (A) ROC curves for adiponectin. (B) ROC curves for insulin. (C) ROC curves for leptin. (D) ROC curves for ALT.

Adiponectin (cut-off ≤ 4.5 μg/mL) had a sensitivity of 85.7 % with a specificity of 79.2 %, while insulin (cut-off ≥ 22 mcUI/mL) had a sensitivity of 78.6 % with a specificity of 79.2 %. A minor discriminatory power was found for: leptin, a cut-off ≥ 36 ng/mL gives a sensitivity of 71.4 % and specificity of 66.7 %, and ALT, a cut-off was ≥ 24 UI/L, with sensibility of 78.6 % e specificity of 73.9 % (Figure 2).

LSM correlated positively with serum levels of insulin, HOMA-IR and both CIMT. Anthropometric, blood pressure, basal insulin, HOMA-IR, leptin, hepatic markers (ALT and GGT) and abdominal fat distribution variables (SAT and VAT) showed positive associations with CAP, while adiponectin showed a negative correlation (Table 3).

**Table 3:** LSM and CAP correlations with cardiometabolic parameters statistically adjusted for BMI.

	LSM (kPa)			CAP (dB/m)		
	r_s_	p	n	r_s_	p	n
BMI Z-Score	0.125	0.45	38	**0.590**	**0.0001**	**38**
AC (cm)	0.359	0.07	26	**0.458**	**0.018**	**26**
WC (cm)	0.337	0.07	28	**0.567**	**0.0016**	**28**
Z SBP	0.134	0.42	38	**0.577**	**0.0001**	**38**
Z DBP	-0.024	0.88	38	**0.569**	**0.0002**	**38**
CIMT Right (cm)	**0.462**	**0.004**	**36**	0.308	0.067	36
CIMT Left (cm)	**0.389**	**0.019**	**36**	0.105	0.54	36
SAT (cm)	0.137	0.42	35	**0.451**	**0.0065**	**35**
VAT (cm)	0.147	0.39	36	**0.574**	**0.0002**	**36**
Insulin (mcUI/mL)	**0.376**	**0.019**	**38**	**0.540**	**0.0005**	**38**
Homa-IR	**0.383**	**0.0174**	**38**	**0.541**	**0.0004**	**38**
Homa-ß	0.189	0.25	38	**0.350**	**0.030**	**38**
TG (mg/dL)	0.204	0.21	38	**0.351**	**0.030**	**38**
Adiponectin (µg/mL)	-0.234	0.15	38	**-0.556**	**0.0003**	**38**
Leptin (ng/mL)	0.218	0.18	38	**0.548**	**0.0004**	**38**
ALT(UI/L)	0.183	0.27	37	**0.519**	**0.001**	**37**
GGT (UI/L)	0.190	0.25	37	**0.468**	**0.003**	**37**
Ferritin (ng/mL)	0.237	0.17	34	**0.371**	**0.030**	**34**

BMI Z-Score, body mass index Z-score; AC, abdominal circumference; WC, waist circumference; SBP, systolic blood pressure; DBP, diastolic blood pressure; CIMT, carotid intima-media thickness; SAT, subcutaneous abdominal tissue; VAT, visceral abdominal tissue; HOMA, homeostatic model assessment; IR, insulin resistance; TG, triglycerides; ALT, alanine aminotransferase; GGT, gamma glutamyl transferase; LSM, liver stiffness measurement; CAP, controlled attenuation parameter.

* non-Gaussian distribution.

## Discussion

This cross-sectional study evaluated differences in a pediatric age group with excess weight related to the presence or absence of MASLD according to transient hepatic elastography. Important metabolic profile differences were observed in patients with MASLD, and also significant correlations were found between the CAP and LSM values and cardiovascular risk factors. It is worth noting that the differences were observed despite both groups presenting patients with previous hepatic enzyme alterations and at high risk for MASLD.

The mean CAP values of the groups with and without steatosis were 288 and 170 dB/m, respectively, Desai et al. demonstrated, in a study where children underwent liver biopsy to confirm the diagnosis of steatosis, that a mean CAP value of 290 dB/m was found in the steatosis group and 198 dB/m for the non-steatosis [16].

There was a significant difference in the LSM patterns when comparing the groups with and without steatosis. Despite the few cases of fibrosis, LSM values correlated with cardiovascular risk factors related to obesity. These results were also found by Gong et al. [17]. and indicate the worsening of the health conditions of these patients related to obesity.

Anthropometric measurements are easy and quick to apply and are considered a good cardiometabolic risk screening, in addition to being related to MASLD [18]. This data support this concept, since the "Steatosis group" was mostly classified as severely obese, and there was a positive association between CAP values (but not with LSM) and anthropometric parameters.

Dysfunctional glucose metabolism plays a key role in the pathophysiology of MASLD [19]. Along with obesity and high triglyceride (TG) levels, a feedback system involving these factors is observed in both adults and children [20]. The present data showed a weak correlation between TG and CAP, higher fasting insulin levels, and greater HOMA-IR values in the "Steatosis group". Unlike Ting et al., who correlated HOMA-IR only with fibrosis [21], the authors found a correlation between insulin resistance markers and both LSM (weak) and CAP values (moderate).

The excess of visceral fat can be considered an important predictor of metabolic syndrome, MASLD, and increased risk for cardiovascular diseases. Despite no consensus for cutoff values to classify cardiovascular risk in children, it is well established that a higher intra-abdominal fat is associated with greater insulin resistance, therefore, the possible presence of MASLD [22]. The present data points in the same direction, as they showed a positive correlation between CAP values and both subcutaneous (SAT) and visceral fat (VAT) indices.

Hypertension can be considered an intersection point between MASLD and metabolic syndrome. A cross-sectional study with a population database demonstrated a link between steatosis through the Hepatic Steatosis Index (HSI), and hypertension in adults [23]. Similar findings were present in this study, with a moderate correlation between CAP levels and SBP Z-score, a supported result of steatosis and cardiovascular risk. Children of the “Steatosis group” could already be investigated for hypertension due to altered BP behavior, which reinforces the need for long-term arterial monitoring of such patients.

Studies describing the association between the results of transient elastography and CIMT are scarce in the literature. However, a cross-sectional study with 96 patients of a specialized medicine center in Italy stated an elevated risk of atherosclerotic disease in patients with fibrosis [24]. This data agrees with the present study, which showed a slightly greater right CIMT in the “Steatosis group” and a positive association was found between LSM and both CIMT.

Adiponectin is recognized as a hormone that plays an important role in regulating homeostasis in healthy individuals. At lower levels, it is associated with metabolic dysfunctions such as obesity and the progression of MASLD, a scenario that can also be observed in children. In the same context, low leptin levels are correlated with appetite regulation and progression of obesity [25,26,27]. Although no studies were found evaluating the relationship between transient elastography and adipokines in children and adolescents, in the present study, CAP values demonstrated moderate correlations, negative with adiponectin and positive with leptin.

The measurement of ALT is used worldwide as a screening for many liver diseases, including MASLD, and can also be related to the severity of the disease. Schwimmer et al. showed that ALT was higher in patients with MASLD when compared to other liver diseases or the control group, being even greater in those with steatohepatitis [28]. To predict the risk of simple steatosis, ROC curves presented the cut-off value of ≥ 22 mcUI/mL to insulin and ≥ 24 IU/L to ALT, for boys and girls.

About the ROC curves for adiponectin and leptin, Boyraz et al. failed to predict the presence or absence of MASLD, according to leptin, but suggested a cut-off value of < 3.2 µg/mL to predict steatosis [29], a value less than ≤ 4.5 µg/mL found in the present study. On the other hand, Manco *et al*. were able to use leptin and TNF-α as predictors of steatohepatitis in children but did not compare groups with or without simple steatosis [30].

## Strengths and limitations

The main limitation of the study was that a liver biopsy was not performed to confirm the diagnosis of MASLD, but it is an invasive test and carries a risk of complications. Although transient elastography is not the gold standard for diagnosing steatosis and fibrosis, many references have considered it a reliable and painless tool for diagnosing and evaluating these conditions in children, viable in research and clinical practice. Another limitation was the small number of participants included, which made it impossible to stratify and evaluate participants by pubertal stage.

On the other hand, transient elastography findings could be compared with several other variables evaluated in MASLD disease, and cross-sectional studies that associate specific parameters of pediatric patients with severe obesity and transient elastography measures (CAP and LSM) are scarce in the literature.

In school-age children and adolescents with excess weight, the presence of steatosis by elastography is related to elevated blood pressure, abdominal obesity, and worse glycemic and hepatic profiles. Furthermore, the present data suggest that low adiponectin and high basal insulin levels are possible biomarkers of MASLD. The study reinforces the need for children with obesity, especially those with a worse metabolic profile, to be investigated for early diagnosis, monitoring, and treatment of MASLD. Further research is needed to confirm these results.

## Funding

This study was partially funded by grants from the Carlos Chagas Filho Foundation for Research Support in the State of Rio de Janeiro (FAPERJ) (APQ1 E-26–111,344-2014, E-26/ 202.866/2015, and E26–201.014–2022, recipient P.S.).

## Data availability statement

The data that support the findings of this study are available from the corresponding author.

## Conflicts of interest

The authors declare no conflicts of interest.
